# Lateral entry into general practice – an explorative analysis of general practice trainees in the competence centre for postgraduate medical education Baden-Württemberg

**DOI:** 10.3205/zma001706

**Published:** 2024-11-15

**Authors:** Jonathan Ko, Katja Krug, Christian Förster, Tanja Jähnig, Martina Bischoff, Christine Becker, Simon Schwill

**Affiliations:** 1 Heidelberg University Hospital, Department of General Medicine and Health Services Research, Heidelberg, Germany; 2 University Hospital Tübingen, Institute for General Medicine and Interprofessional Care, Tübingen, Germany; 3 University Hospital Ulm, Institute for General Medicine, Ulm, Germany; 4 University Medical Center Freiburg, Institute of General Medicine, Freiburg, Germany

**Keywords:** general practice, family medicine, postgraduate medical education, lateral entry, competence centres

## Abstract

**Objective:**

To aid the shortage of general practitioners (GPs) in Germany, since 2011 medical specialists from other fields may switch specialisation by undergoing a slim-lined training programme (lateral entry) into general practice (GP). Available published qualitative and quantitative data on lateral entrants (LEs) is scarce. Aim of the study was an explorative analysis of LEs in the competence centre for postgraduate medical education Baden-Wuerttemberg (KWBW).

**Methods:**

In 2016, a multicentric cohort study was initiated including all GP trainees entering the KWBW. Data from 2016 to 2022 was included (T0). A follow-up with graduates from the KWBW was performed once in 2023 (T1). Surveys at T0 and T1 were self-developed, piloted, and included questions on social demography, education, qualification, current training, working environment and professional plans. Dataset was analysed exploratively.

**Results:**

At T0, 884 GP trainees participated (response 95.2%). 23.8% of participants were LEs (N=210). Most LEs were specialists from anaesthesiology (34.8%), internal medicine (28.5%) and surgery (20.6%). LEs had been working in their previous specialty for a median of 3 years before starting GP-training. At T0, LEs were seven years older than their colleagues (p<0,001). The proportion of men among LEs was higher (34.3% vs 25.1%, p=0,009). LEs were more decisive to open their own GP practice (p=0,009). At T1, 48 LEs participated of which 92% were practising GPs (N=36). 64,5% considered themselves rural GPs and work in communities <20.000 people (N=36). LEs mainly choose GP because of its specific way of working, job dissatisfaction and personal motives such as opportunity to start a practice. Most LEs strongly agreed that they would switch to GP again.

**Discussion:**

LEs are a relevant party among GP trainees. Many LEs become self-employed and contribute essentially to providing primary care. Lateral entry attracts specialists, also from rural areas, seeking for professional satisfaction. This is why LEs should follow their new path within a regional competence centre providing GP specific courses, mentoring and a professional network.

**Conclusions:**

LEs graduating from KWBW have become an important pillar of primary care in Baden-Wuerttemberg.

## Zusammenfassung

**Zielsetzung:**

Um dem Mangel an Hausärzt*innen in Deutschland entgegenzuwirken, können seit 2011 Gebietsspezialisten durch eine verkürzte Weiterbildung in die Allgemeinmedizin wechseln (=Quereinstieg). Bisher gibt es nur wenige veröffentlichte qualitative und quantitative Daten zu Quereinsteiger*innen (QE). Ziel der Studie war eine explorative Analyse von QE im Kompetenzzentrum Weiterbildung Baden-Württemberg (KWBW) durchzuführen.

**Methoden:**

2016 wurde eine multizentrische Kohortenstudie mit Ärzt*innen in Weiterbildung (ÄiW) Allgemeinmedizin im KWBW initiiert. Es wurden Daten von 2016 bis 2022 einbezogen (T0). Ein Follow-up mit Absolventen des KWBW wurde im Jahr 2023 durchgeführt (T1). Die Fragebögen zu T0 und T1 wurden selbst entwickelt und pilotiert und enthielten Fragen zur Soziodemographie, Ausbildung, Qualifikation, aktuellen Weiterbildung, zum Arbeitsumfeld und zu beruflichen Plänen. Der Datensatz wurde explorativ ausgewertet.

**Ergebnisse:**

Zu T0 nahmen 884 ÄiW an der Studie teil (Rücklauf 95,2%). 23,8% der Teilnehmenden waren QE (n=210). Die meisten QE waren Fachärzt*innen für Anästhesiologie (34,8%), Innere Medizin (28,5%) und Chirurgie (20,6%). QE hatten im Durchschnitt 3 Jahre in ihrem vorherigen Fachgebiet gearbeitet, bevor sie die Weiterbildung Allgemeinmedizin begannen. Zum Zeitpunkt T0 waren QE sieben Jahre älter als ihre Kolleg*innen (p<0,001). Der Anteil der Männer unter den QE war höher (34,3% gegenüber 25,1%, p=0,009). QE waren eher entschlossen, eine eigene Hausarztpraxis zu eröffnen (p=0,009). An T1 nahmen 48 QE teil, von denen 92% hausärztlich tätig waren (n=36). 64,5% betrachteten sich als Landärzt*in und arbeiteten in Gemeinden mit weniger als 20.000 Einwohnern (n=36). QE entschieden sich hauptsächlich für die Allgemeinmedizin aufgrund der spezifischen Arbeitsweise, beruflicher Unzufriedenheit und aus persönlichen Gründen, wie z. B. der Möglichkeit, sich niederzulassen. Die meisten QE stimmten nachdrücklich zu, dass sie wieder in die Allgemeinmedizin wechseln würden.

**Diskussion:**

QE sind eine wichtige Gruppe unter den ÄiW in der Allgemeinmedizin. Viele QE machen sich selbstständig und tragen wesentlich zur Primärversorgung bei. Der Quereinstieg zieht Gebietsspezialisten an, auch aus ländlichen Gebieten, die nach beruflicher Erfüllung suchen. Deshalb sollten QE auf ihrem neuen Weg von einem Kompetenzzentrum vor Ort unterstützt werden, das hausarztspezifische Kurse, Mentoring und ein berufliches Netzwerk anbietet.

**Schlussfolgerung:**

QE, die das KWBW durchlaufen haben, sind zu einer wichtigen Säule der Primärversorgung in Baden-Württemberg geworden.

## Introduction

In the past decades the predicted shortage of General Practitioners (GPs) in Germany has become increasingly evident [1], [2]. On the one hand this is due to a quantitative lack of GPs resulting from an insufficient number of newly qualified GPs in a system that allows junior doctors to freely choose the specialty they want to be trained in [3]. This is further driven by an aging GP workforce as in the next few years up to a third of GPs will enter retirement age [1], [4]. At the same time data has shown that more and more GPs are working part-time thereby reducing the overall care that can be provided [4], [5]. On the other hand, the German population is ageing and will therefore require more medical care due to the higher prevalence of multimorbidity [6].

To tackle the shortage of GPs in the midterm as well as establishing a standardised and coordinated postgraduate medical training programme, competence centres (*Kompetenzzentren*) for GP-training were established in the different states in Germany. The KWBW-Verbundweiterbildung*^plus®^* in the state of Baden-Wuerttemberg is based on the Verbundweiterbildung*^plus® ^*Baden-Wuerttemberg founded in 2008 [7], [8].

To recruit GPs in the short term, the German Medical Association (*Bundesärztekammer*) has issued a letter of recommendation in 2011 which allows medical specialists from other fields to change to GP more easily by undergoing a slim-lined training programme called lateral entry (*Quereinstieg*) [9]. According to the most recent postgraduate training regulations of 2020, a regular GP trainee in Baden-Württemberg must complete 12 months in internal medicine in hospital, 24 months in a GP clinic, 6 months in a different specialty (e.g. surgery) and 18 months can be chosen ad libitum [10]. For lateral entrants (LEs) their previous specialist training is recognised as 36 months and they must further train for 24 months in GP. In the state of Baden-Württemberg lateral entry has been implemented in 2011 [11].

Since the introduction of lateral entry into family medicine there has only been little research in this matter. A qualitative study by Schwill et al. highlights the motives of LEs, which are mainly the wish to intensify the quality of patient contacts, self-employment opportunities as well as feelings of frustration over poor working conditions in hospitals [12]. Another study by Syrieyx et. al. with N=64 points out that the shortened training programme in lateral entry motivated specialists to switch into GP [13]. 

We aimed to describe physicians that choose lateral entry in comparison to regular GP trainees (NLEs). Additionally, we intended to explore the career development of LEs who have completed their postgraduate GP training. 

## Methods

### Study design

This study incorporates a multicentric cross-sectional survey as a starting point of a cohort study with data from 2016-2022 (T0) and a cross-sectional follow-up survey with GP trainees in lateral entry in 2023 (T1). 

### Setting

The KWBW-Verbundweiterbildung*^plus®^* is a voluntary postgraduate medical training programme provided by the General Practice Departments of the Universities of Freiburg, Heidelberg, Tübingen and Ulm and supported by the *Landesaerztekammer Baden-Wuerttemberg*, the *Kassenaerztliche Vereinigung Baden-Wuerttemberg* and the *Baden-Wuerttembergische Krankenhausgesellschaft*. It is a voluntary programme for junior doctors in GP training that consists of seminars, networking opportunities, mentoring and the possibility to participate in regional rotation programmes. Additionally, the KWBW offers train the trainer courses for GP trainers [14]. The KWBW-Verbundweiterbildung*^plus®^* is also available to LEs.

### Participation and recruitment

Prerequisite for participation in the survey upon entry was enrolment in the KWBW. Recruitment for T0 took place during the mandatory two-day introductory events (online or face-to-face). Prerequisite for participation in the follow-up at T1 is completion of the programme and successful board certification in GP. 

### Instrument development

A standardised German instrument for a comprehensive survey of new attendees of a postgraduate medical training programme or qualified GPs was not available. Therefore, an interprofessional, multicentric project team from the departments of General Practice of the Universities of Freiburg, Heidelberg, Tübingen and Ulm familiar with the target groups designed a survey based on literature review and assessment of content relevant to the target groups. Primary validation was performed using the think-aloud technique with five junior doctors (basic survey at T0) and two junior doctors and two GPs (Follow up at T1) familiar with the training programme. The basic survey at T0 has been slightly adapted several times since the beginning of the study (version 1: 28.01.2016; version 2: 03.03.2016-17.03.2016; version 3: 16.06.2016-15.03.2018; version 4: 15.03.2018; version 5: since 05.04.2018). Changes to the instrument included reduction of free-text-answers and reduction of items to support accelerated standardized analysis. The different motives of specialists that have previously been explored in a qualitative study by Schwill et al. in 2016 [12] were integrated for quantitative confirmation. 

### Instrument description

The basic survey at T0 consisted of a total of 62 questions. There were 7 subsections including social demographics, medical education, qualifications, postgraduate medical training, future planning for postgraduate medical training, future planning as a specialist and completed postgraduate training. The follow-up at T1 consists of a total of 109 questions, with varying number of questions shown to participants depending on professional background and current occupation. Both surveys are composed of binary response options, free text statements, multiple choice questions and preference statements on a seven or six-point Likert scale (1=not at all important, 6/7=very important). 

### Conducting the survey

The basic survey at T0 was presented to all participants at introductory seminars of the KWBW between 2016 and 2022. The survey was completed voluntarily and without time constraints. From 2016 to 2021 the survey was paper-based and since 3^rd^ December 2021 it is being conducted online (Survey Monkey^®^, San Mateo, CA, USA).

The follow-up at T1 was conducted online (Survey Monkey^®^, San Mateo, CA, USA) via email on 27^th^ July 2023. The survey was completed voluntarily. Two email reminders were sent in August and September 2023, as well as a reminder letter by post on 8^th^ September 2023. The survey was closed on 10^th^ October 2023. 

### Data analysis

Data analysis was performed using IBM^®^ SPSS^®^ Statistics version 29.0 for Windows (IBM, Armonk, NY, USA). Dataset was described descriptively. If applicable, t-test, Mann-Whitney-U-test and Pearson-Chi-square-test were used for comparative analysis. 

### Ethics

All participants gave written consent to participate in the study and to the pseudonymised use of their data. Ethical approval by the ethics-commission in Heidelberg was granted (No. S-570/2015).

## Results

### Participation at T0

From 2016 to 2022 n=884/929 GP trainees that enrolled in the KWBW training programme participated in the basic survey at T0 (response rate: 95.2%), of which 23.8% (n=210) were LEs.

### Social demographics at T0

An overview of the social demographics as well as comparison between NLEs and LEs at T0 can be found in attachment 1 .

77.5% (n=138) of the LEs have partners with a university degree, 37.6% (n=67) of them are physicians as well, which is a significantly higher proportion than NLEs (p=0.02). On average LEs had taken 12 months of parental leave prior to lateral entry (Q1;Q3 2.5;27; Mean [SD]: 18 [1.54]). 26.8% (n=56) of LEs grew up in a city (population count >100.000), 23.4% (n=49) in a town (20.001-100.000), 41.7% (n=87) in a small town (5.000-20.000) and 8.1% (n=17) in a village (<5.000). For those that did not grow up in a city, 5.3% (n=11) lived less than 10km, 35.1% (n=73) lived 10-35km and 34.6% (n=72) lived >35km away from the next city. Compared to NLEs, LEs grew up in places further away from cities (p=0,044) but there was no significant difference in the population size of their hometowns. There were no significant differences between NLEs and LEs concerning their performances in education, such as the average grade in high school (“Abitur”, German A-levels), their final grade in their medical education and publication records.

### Postgraduate training at T0

Compared to NLEs (87.8%; n=589), less LEs have worked in internal medicine (45.7%; n=96; p<0.001) while having rotated more often to the department of anaesthesiology (40.5%; n=85; p<0.001) and surgery (26.7%; n=56; p=0.039). In relation to NLEs (5.2%; n=35) more LEs were additionally qualified in emergency medicine (39.5%; n=83; p<0.001). 

### Future plans concerning postgraduate training at T0

Compared with NLEs, LEs prefer to have less rotations and finish their postgraduate training at one post (p<0.001). There was no significant difference between NLEs (71.8%; n=475) and LEs (66.7%; n=136) concerning their preference to have a training post in a rural practice. More LEs preferred to work full-time (57.6%; n=118) compared to NLEs (47.9%; n=317; p=0.05). LEs stated (on a scale from 1-7; 1=not important at all; 7=very important) that the prospect of continuing to be employed by the same practice after qualifying as GP (mean: 4.46 (SD: 2.05); Md: 5 (Q1: 3; Q3: 6)) was more important to them as compared to NLEs (mean: 4.08 (SD: 1.92); Md: 4 (Q1: 2; Q3: 6); p=0.008). Similarly, it was more important to LEs (same scale as above; mean: 4.22 (SD:1.99); Md: 4 (Q1: 2; Q3: 6)) than NLEs (mean: 3.45 (SD: 1.97); Md: 4 (Q1: 2; Q3: 5)) to have the chance to become a partner of the practice (p<0.001).

### Future plans as a qualified GP at T0

By comparison, LEs (45.2%; n=94) were more decisive to open their own GP practice and work self-employed than NLE (35.3%; n=234; p=0,009). Concerning the preferred size of the place of residence, distance of the place of residence to the next city, working in a rural practice or acquiring additional qualifications there were no significant differences between LEs and NLEs (see attachment 1 ). 

### Results at T1

An overview of the results at T1 in comparison to LEs at T0 are shown in table 1 (Tab. 1), further results on LEs are shown in table 2 (Tab. 2). LEs at T1 were more likely working in smaller communities: n=8 (2.8%) <5.000 inhabitants, n=15 (41.7%) 5.000-20.000 inhabitants, N=4 in cities with >100.000 inhabitants. 97.2% (n=35) were working in Baden-Wuerttemberg and 47.2% (n=17) were working more than 40 hrs/week.

## Discussion

To the best of our knowledge this explorative analysis of LEs is the largest quantitative study on LEs, showing typical attributes of lateral entrants and highlighting their importance in the effort to acquire more GPs in Germany. 

### Typical attributes of lateral entrants

In comparison to NLEs the average LE is more likely to be older, married, to have more children and a Dr. med.-degree. After all, these findings are not surprising due to the very nature of LE taking place later in one’s career and life. It is important to note that among LEs the proportion of men is significantly higher than the proportion of men among NLEs. Nevertheless, in both subgroups women make up the majority. This is in line with the general trend of more women going into medicine [15] and especially into GP [1], [16], [17], [18]. Most LEs who changed to GP are specialists in anaesthesiology, internal medicine and surgery (including orthopaedics and trauma surgery). Inferring from the motives of LEs as described by Schwill et al. [12] it appears that with some professional experience long-term patient-oriented care as well as self-employed work are becoming more attractive. Otherwise, working conditions and career prospects in various specialties are not attractive enough to retain colleagues slightly interested in GP. Recent studies in the fields of anaesthesiology, internal medicine and surgery share this observation and have shown a high degree of dissatisfaction among junior doctors concerning their working and training situation [19], [20], [21]. This has mainly been attributed to long working hours, a lack of compatibility of family and work and an increasing burden of administrative work. Analyses of LEs by the German Medical Association (*Bundesärztekammer*) are not accessible. 

### Lateral entrants make up considerable amount of GP trainees

Currently nearly one quarter of all participants of the KWBW are LEs. They need to be acknowledged as a substantial and distinct subgroup among GP trainees. Therefore, postgraduate training programmes such as the KWBW should consider their needs and offer appropriate activities, such as LE-targeted mentoring sessions. As a pitfall reported by LEs (data not shown), LEs might get caught in a double role as qualified specialists while being GP trainees as well. For this reason, they might only be treated as medical specialists and not as trainees that require teaching and training. To prevent this, it is important to raise awareness both among GP trainers as well as LEs themselves. GP trainers could be sensitized in train-the-trainer courses that involve modules on the specific characteristics and needs of LEs. Up to date, competence centers have already been providing various networking opportunities for GP trainees (including LEs) which often prompt them to reflect about their (potentially conflicting) double roles as service providers and doctors in training. LEs that take part and engage in these networking opportunities including group-mentoring benefit especially due to their professional experience

From the KWBW experience, GP-trainers need support to reflect their own views on LEs and to accept their obligation to induce and accompany a sound change of specialty among LEs, even in the first years after training. At the same time, awareness among LEs should be induced: Every GP trainee (LEs as well as non-LEs) entering the KWBW-Verbundweiterbildung*^plus®^* start with a compulsory two-day training day about the values and core principles of primary care and family medicine. In these courses the attendants reflect on their own views and presumptions of family medicine. From our experience not only the LEs do learn about the core principles the first time (data not published yet). This highlights the importance for LEs to participate in a training program such as the KWBW-Verbundweiterbildung*^plus®^*. 

### Lateral entrants – a pillar of family medicine

LEs are a current and future pillar of GP in Baden-Wuerttemberg/Germany. Currently there is a trend among young physicians (including GP trainees) towards working part-time and away from self-employment [22]. Our study reveals that the largest part of LEs end up working in family medicine. Moreover, many LEs are living in rural areas and consider themselves as rural GPs, thereby contributing to patient care in physician shortage areas. It needs to be noted, that there was no significant difference between NLEs and LEs in terms of their future plans to work in a rural practice. Most LEs do not regret having changed to family medicine which indicates a high level of satisfaction and motivation to work as GPs. As an opportunity, with their previous expertise such as emergency medicine, LEs may help broaden the skills of GPs to provide comprehensive primary care. Finally, nearly half of the LEs become self-employed, while further surveys have yet to reveal the long-term trend in terms of self-employment. LEs - especially from hospital - are attracted by the working-profile and working-conditions of GP, “economic motives” do not seem to play a significant role in their decision. Regarding (future) problems of GP in Germany, LEs form a resource to deal with shortage of GPs. However, some regional medical association are discussing to cut down the duration of lateral entry from 2 to 1 year [23]. Perhaps, the beneficial 2-year experiences of LEs in the KWBW need to be promoted even more among physicians working in specialisation. 

### Limitations

Data about graduates of LE in neither Baden-Wuerttemberg nor Germany are accessible, which is why we are not sure how many LEs are considered in the study. However, 40% of GP trainees in Baden-Wuerttemberg enter the KWBW and the number of LEs is comparably high. Participants of the KWBW appear as a selected group among GP trainees, more likely with a higher motivation to participate in additional training (selection bias). Finally, intraindividual follow-up from T0 to T1 was provided by pseudonym, but due to the low number of matches (n=15) we refrained from conducting further analyses with the matched pairs. Further reminders and continuous surveys are necessary to gain sufficient responses. 

## Conclusions

Our study is an important quantitative study on LEs in Germany because LEs among GP trainees are numerous. Most differences between LEs and NLEs might be due to their more advanced age. LE attracts specialists from the hospital sector. Many LEs become self-employed and provide primary care in rural areas. With their high degree of determination to continue working as GPs and unique background they have become an important pillar of primary care in Baden-Württemberg. Overall, lateral entry seems to be an effective way in easing the pressure on primary care by encouraging more physicians to change to GP. However, prerequisite for a successful LE is the duration of 2 years in GP as well as the enrolment in a competence centre providing core principles of GP and offering a strong collegial network to LEs. 

## Authors’ ORCIDs

- Katja Krug: [0000-0002-9795-7954]
- Christian Förster: [0000-0001-7068-2084]
- Simon Schwill: [0000-0002-0954-2194]

## Acknowledgements

We would like to thank Dr. Viviane Deugoué, Dr. Dorothee Reith, Dr. Sandra Stengel and Mr. Tobias Walter for their support. We would also like to thank the entire KWBW team, all seminar moderators, trainers, facilitators and participants of the KWBW Verbundweiterbildung*^plus®^* for their inspiring joy in teaching as well as learning, commitment and willingness to further develop the programme. We would like to thank all our cooperation partners for their valuable support.

It was challenging to find an appropriate English term for “Quereinstieg” (German). To our knowledge there is no equivalent training-programme in other countries, only very few publications on this topic in general and no established terminology in English. After considering other alternatives (such as “short-track” or “cross-entry”) we favoured “lateral entry” since in our opinion it captures the meaning of “Quereinstieg” the best. Furthermore, it has been used in the same context in another recent publication (2022 Syrieyx). 

## Competing interests

The authors declare that they have no competing interests. 

## Supplementary Material

General Practice Trainees of the competence centre for postgraduate medical education Baden-Wuerttemberg (KWBW): Comparison at programme start between non-lateral-entrants and lateral entrants (2016–2022)

## Figures and Tables

**Table 1:**
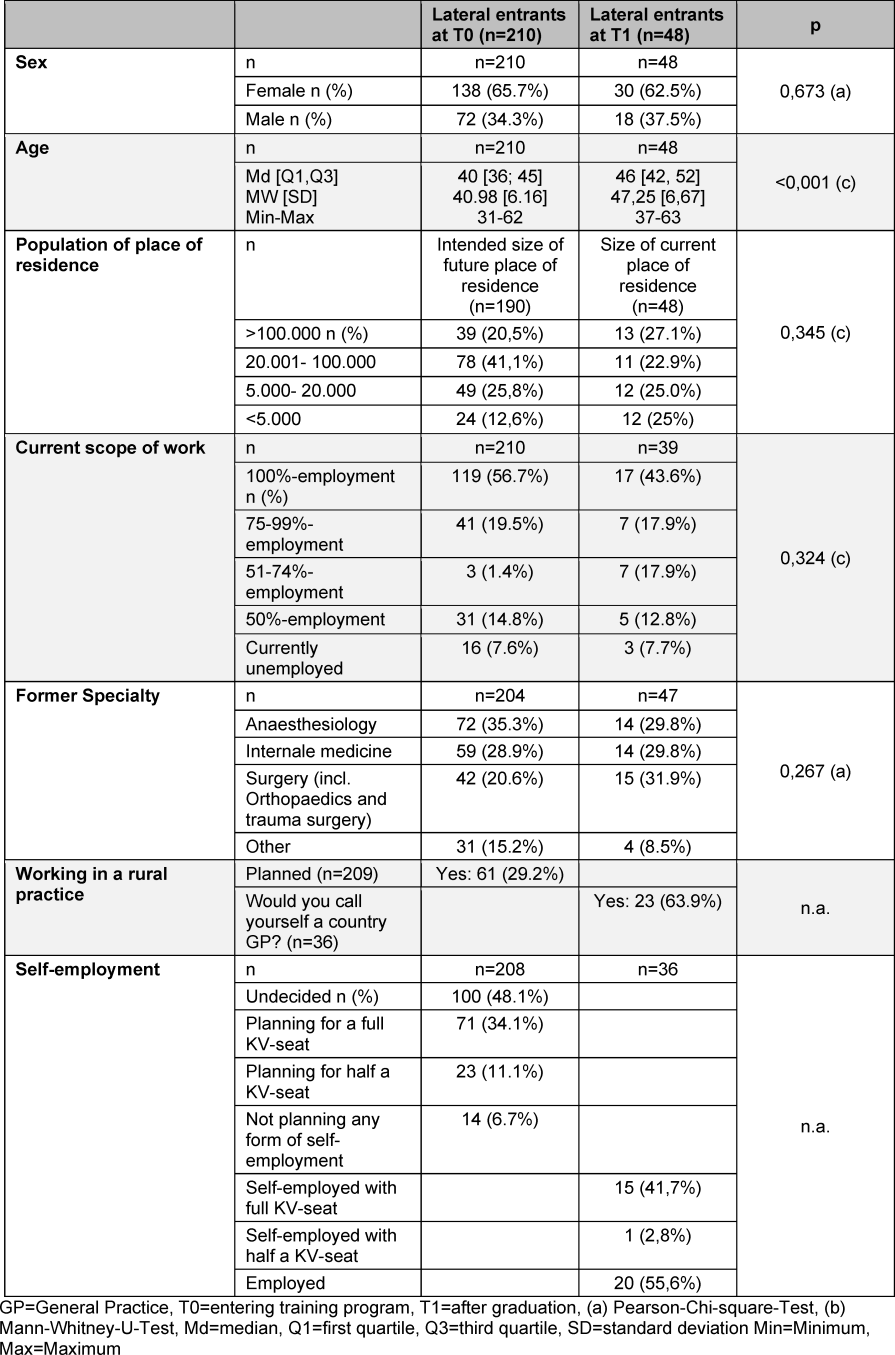
General practice trainees of the competence centre for postgraduate medical education Baden-Wuerttemberg (KWBW): Lateral entrants (LE) at T0 and T1

**Table 2:**
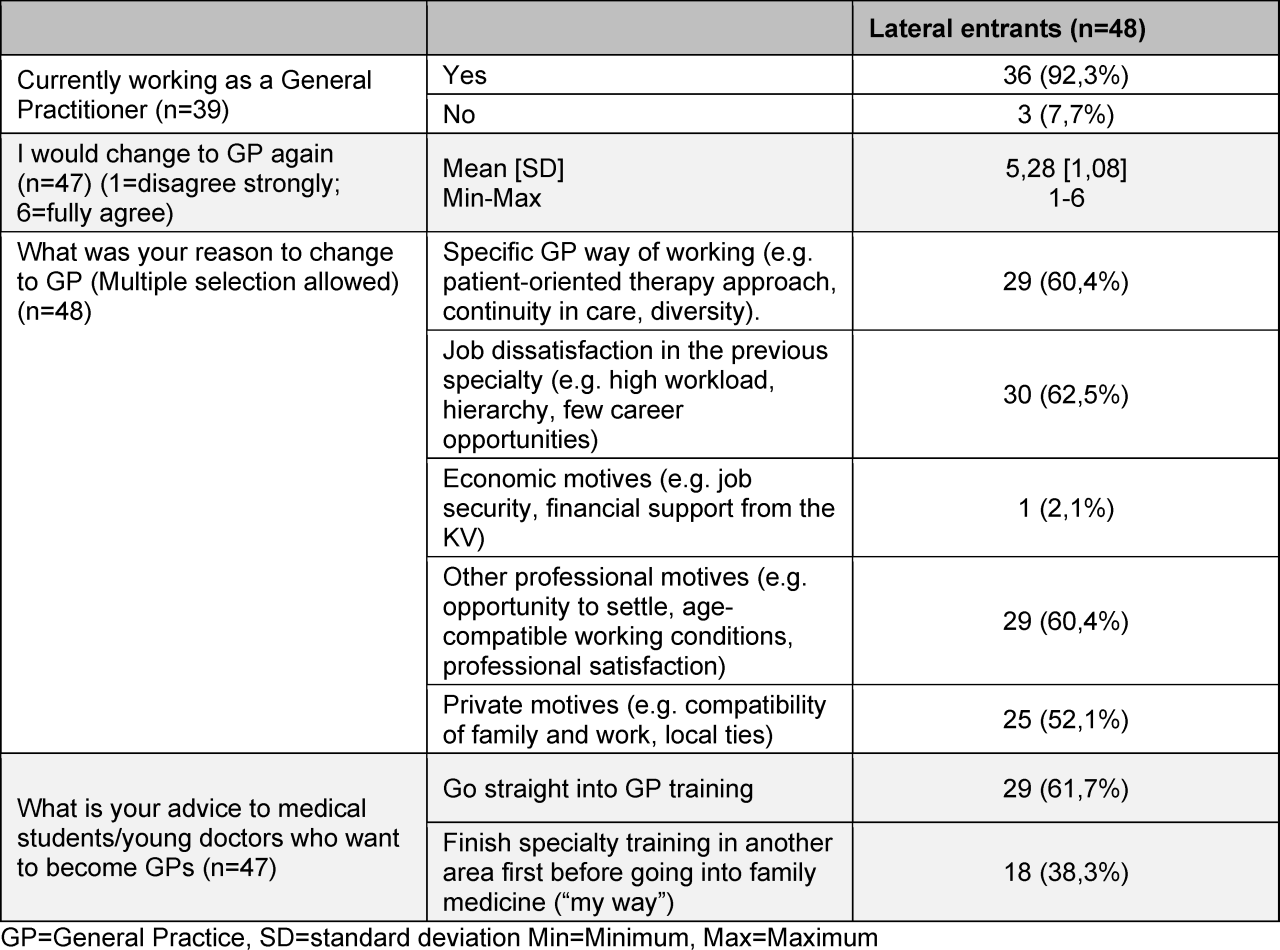
General practice trainees of the competence centre for postgraduate medical education Baden-Wuerttemberg (KWBW): Further results of Lateral entrants T1 (after graduation)
