# Supplementary material for: Lateral entry into general practice – an explorative analysis of general practice trainees in the competence centre for postgraduate medical education Baden-Württemberg
Source: GMS J Med Educ. 2024 Nov 15;41(5):Doc51. doi: 10.3205/zma001706 (PMC11656172; doi:10.3205/zma001706)
Supplement: General Practice Trainees of the competence centre for postgraduate medical education Baden-Wuerttemberg (KWBW): Comparison at programme start between non-lateral-entrants and lateral entrants (2016–2022) [file JME-41-51-s-001.pdf]

Attachment 1: General Practice Trainees of the competence centre for postgraduate medical education Baden-Wuerttemberg (KWBW): Comparison at programme start between non-lateral-entrants and lateral entrants (2016 - 2022)

		All GP Trainees (n=884)	Non-lateral-entrants (NLE) (n=674,76.2%)	Lateral entrants (LE) (n=210, 23.8%)	p (between NLE and LE)
**Social demographics**					
**Sex**	n	n=883	n=672	n=210	0.009 (a)
	Female n (%)	642 (72.6%)	503 (74.9%)	138 (65.7%)	
	Male n (%)	241 (27.3%)	169 (25.1%)	72 (34.3%)	
**Age**	n	n=880	n=669	n=210	<0.001 (b)
	Md [Q1,Q3]<br>MW [SD]<br>Min-Max	34 [31;38]<br>35.5 [6.31]<br>24-65	33 [30; 36]<br>33.77 [5.3]<br>24-65	40 [36; 45]<br>40.98 [6.16]<br>31-62	
**Relationship Status**	n	n=881	n=671	n=209	<0.001 (a)
	Single n (%)	216 (24,4%)	185 (27,6%)	31 (14,8%)	
	Firm partnership	525 (59,4%)	379 (56,3%)	145 (69,4%)	
	Married	216 (24,4%)	185 (27,6%)	31 (14,8%)	
**Parenthood**	n	n=884	n=673	n=210	<0.001 (b)
	No. of parents n (%)	503 (56.9%)	342 (50.8%)	160 (76.2%)	
	No. of children<br>Md [Q1;Q3]<br>Min-Max	1 [0;2]<br>0-5	1 [0; 2]<br>0-5	2 [1; 2]<br>0-4	
**Immigration background**	n	n=884	n=673	n=209	0.012 (a)
	With immigration background n (%)	198 (22.4%)	157 (23.3%)	41 (19.5%)	
**Studies and qualifications**					
**High school grade (Abitur. German A-levels)**	n	n=861	n=662	n=198	0.18 (b)
	Mean [SD]	1,66 [0.54]	1.65 [0.53]	1.72 [0.58]	
**Final grade medical school (n=784)**	n	n=784	n=612	n=172	0.282 (b)
	Mean [SD]	2.15 [0.56]	2.16 [0.56]	2.13 [0.59]	
**Medical school in Baden-Württemberg**	n	n=883	n=673	n=209	n.s.
	n (%)	510 (57.8%)	386 (57.4%)	124 (59.3%)	
**Completion of medical dissertation (Dr. med.)**	n	n=875	n=577	n=192	<0.001 (a)
	Dissertation completed n (%)	475 (54.3%)	102 (17.7)	146 (76.0%)	

Attachment 1 to Ko J, Krug K, Förster C, Jähnig T, Bischoff M, Becker C, Schwill M. *Lateral entry into General Practice – An explorative analysis of General Practice trainees in the competence centre for postgraduate medical education Baden-Württemberg*. GMS J Med Educ. 2024;41(5):Doc51. DOI: 10.3205/zma001706

**Publication record**	n	n=873	n=666	n=207	0.503 (a)
	First author n (%)	92 (10.5%)	66 (9.9%)	26 (12.6%)	
	Co-author	244 (27.9%)	185 (27.8%)	59 (28.5%)	
**Current scope of work (n=883)**	n	n=883	n=673	n=210	0.375 (b)
	100%-employment n (%)	492 (55.7%)	373 (55.4%)	119 (56.7%)	
	75-99%-employment	146 (16.5%)	105 (15.6%)	41 (19.5%)	
	51-74%-employment	15 (1.7%)	12 (1.8%)	3 (1.4%)	
	50%-employment	137 (15.5%)	106 (15.8%)	31 (14.8%)	
	Currently unemployed	93 (10.5%)	77 (11.4%)	16 (7.6%)	
**Popluation in current place of residence**	n	n=877	n=668	n=208	0.35 (b)
	>100.000 n (%)	309 (35.2%)	247 (37.0%)	61 (29.3%)	
	20.001- 100.000	253 (28.8%)	178 (26.6%)	75 (36.1%)	
	5.000- 20.000	270 (30.8%)	208 (31.1%)	62 (29.8%)	
	<5.000	45 (5.1%)	35 (5.2%)	10 (4.8%)	
**Current stage of post-graduate training**	n	n=865	n=660	n=205	<0.001 (a)
	Outpatient n (%)	633 (73.2%)	432 (65.5%)	201 (98.0%)	
	In-hospital n (%)	232 (26.8%)	228 (34.5%)	4 (2.0%)	
**Future Plans**					
**Preferred state of residence after specialty exam**	n	n=861	n=655	n=205	n.s.
	Baden-Württemberg n (%)	791 (91.9%)	593 (90.5%)	197 (96.1%%)	
**Preferred size of long-term workplace (population)**	n	n=795	n=602	n=192	0.402 (b)
	>100.000 n (%)	121 (15.2%)	100 (16.6%)	21 (10.9%)	
	20.001-100.000	282 (35.5%)	206 (34.2%)	76 (39.6%)	
	5.000-20.000	288 (36.2%)	220 (36.5%)	68 (35.4%)	
	<5.000	104 (13.1%)	76 (12.6%)	27 (14.1%)	
**Working in a rural practice (n=877)**	n	n=877	n=667	n=209	0.81 (a)
	Imaginable n (%)	706 (80.5%)	538 (80.7%)	167 (79.9%)	
	N	n=543	n=414	n=129	0.915 (a)
	Planned n (%)	259 (47.7%)	198 (47.8%)	61 (47.3%)	
**Area of work after specialty exam**	n	n=866	n=659	n=206	0.055 (a)
	General Practice n (%)	847 (97.8%)	641 (97.3%)	205 (99.5%)	
	Outside of General Practice	19 (2.2%)	18 (2.7%)	1 (0.5%)	

Attachment 1 to Ko J, Krug K, Förster C, Jähnig T, Bischoff M, Becker C, Schwill M. *Lateral entry into General Practice – An explorative analysis of General Practice trainees in the competence centre for postgraduate medical education Baden-Württemberg*. GMS J Med Educ. 2024;41(5):Doc51. DOI: 10.3205/zma001706

**Preferred place of work after specialty exam** (Multiple mentionings possible)	n	n=881	n=670	n=210	
	Single practice n (%)	146 (16.6%)	110 (16.4%)	35 (16.7%)	0.932 (a)
	Joint practice	341 (38.7%)	238 (35.5%)	103 (49.0%)	<0.001 (a)
	Practice sharing	627 (71.2%)	486 (72.5%)	140 (66.7%)	0.101 (a)
	Community health centre	300 (34.1%)	228 (34.0%)	71 (33.8%)	0.953 (a)
	Research department at university	60 (6.8%)	48 (7.2%)	12 (5.7%)	0.467 (a)
**Intended self-employment**	n	n=872	n=663	n=208	0.009 (a)
	Undecided n (%)	462 (53.0%)	361 (54.4%)	100 (48.1%)	
	Yes, full time	218 (25.0%)	147 (22.2%)	71 (34.1%)	
	Yes, half time	110 (12.6%)	87 (13.1%)	23 (11.1%)	
	Self-employment is ruled out	79 (9.1%)	65 (9.8%)	14 (6.7%)	
	Private doctor	3 (0.3%)	3 (0.5%)	0	

GP=General Practice, (a) Pearson-Chi-Quadrat-Test, (b) Mann-Whitney-U-Test, Md=median, Q1=first quartile, Q3=third quartile, SD=standard deviation, Min=Minimum, Max=Maximum
